# Prenatal PM_2.5_ affects atopic dermatitis depending on maternal anxiety and gender: COCOA study

**DOI:** 10.1002/clt2.12070

**Published:** 2021-10-15

**Authors:** Sangrok Kim, Song‐I Yang, Hyeyeun Lim, So‐Yeon Lee, Min Jee Park, Kun‐Baek Song, Eom Ji Choi, Hea Young Oh, Hwan‐Cheol Kim, Yee‐Jin Shin, Kyung‐Sook Lee, Kil Yong Choi, Dong In Suh, Youn Ho Shin, Kyung Won Kim, Kangmo Ahn, Soo‐Jong Hong

**Affiliations:** ^1^ Department of Medical Science Asan Medical Institute of Convergence Science and Technology Asan Medical Center Ulsan University College of Medicine Seoul Republic of Korea; ^2^ Department of Pediatrics Hallym University Sacred Heart Hospital Hallym University College of Medicine Anyang Republic of Korea; ^3^ Department of Pediatrics Childhood Asthma Atopy Center Humidifier Disinfectant Health Center Asan Medical Center University of Ulsan College of Medicine Seoul Republic of Korea; ^4^ Department of Pediatrics Uijeongbu Eulji Medical Center Uijeongbu Republic of Korea; ^5^ Department of Medicine Asan Medical Center Ulsan University College of Medicine Seoul Republic of Korea; ^6^ Department of Occupational and Environmental Medicine Inha University School of Medicine Incheon Republic of Korea; ^7^ Department of Psychiatry Yonsei University College of Medicine Seoul Republic of Korea; ^8^ Department of Rehabilitation Hanshin University Osan Republic of Korea; ^9^ Department of Environmental Energy Engineering Anyang University Anyang Republic of Korea; ^10^ Department of Pediatrics Seoul National University College of Medicine Seoul Republic of Korea; ^11^ Department of Pediatrics CHA Gangnam Medical Center CHA University School of Medicine Seoul Republic of Korea; ^12^ Department of Pediatrics Yonsei University College of Medicine Seoul Republic of Korea; ^13^ Department of Pediatrics Samsung Medical Center Sungkyunkwan University School of Medicine Seoul Republic of Korea

**Keywords:** anxiety, atopic dermatitis, gender, PM_2.5_, prenatal

## Abstract

**Background:**

The prevalence of atopic dermatitis (AD) is increasing worldwide. Prenatal particulate matter with an aerodynamic diameter <2.5 μm (PM_2.5_) and maternal anxiety during pregnancy has been suggested as a potential causes of AD. This study investigated the effects of prenatal PM_2.5_ and maternal anxiety on AD and identified the critical period of PM_2.5_ exposure for AD in infants.

**Methods:**

This study included 802 children from the COCOA birth cohort study with follow‐up data at 1 year of age. PM_2.5_ was estimated by land‐use regression models and prenatal anxiety was measured with a questionnaire. AD was diagnosed by doctor at 1 year of age. Logistic regression analysis and Bayesian distributed lag interaction models were applied.

**Results:**

Higher PM_2.5_ during the first trimester of pregnancy, higher prenatal maternal anxiety, and male gender were associated with AD at 1 year of age (adjusted odds ratio [aOR] and 95% confidence interval [CI]: 1.86 [1.08–3.19], 1.58 [1.01–2.47], and 1.54 [1.01–2.36], respectively). Higher PM_2.5_ during the first trimester and higher maternal anxiety during pregnancy showed an additive effect on the risk of AD (aOR: 3.13; 95% CI: 1.56–6.28). Among boys exposed to higher maternal anxiety during pregnancy, gestational weeks 5–8 were the critical period of PM_2.5_ exposure for the development of AD.

**Conclusions:**

Higher PM_2.5_ exposure during gestational weeks 5–8 increased the probability of AD in infancy, especially in boys with higher maternal anxiety. Avoiding PM_2.5_ exposure and maternal anxiety from the first trimester may prevent infant AD.

## 1 INTRODUCTION

Atopic dermatitis (AD) is a chronic inflammatory skin disease occurring mainly in infants.^1^ The prevalence of AD in children has been increasing worldwide and in Korea.^2^ Although the cause of AD has not been clearly identified, genetic and environmental factors have a role.^1^, ^2^ Among several environmental factors, particulate matter with an aerodynamic diameter of less than 2.5 μm (PM_2.5_) and maternal distress during pregnancy are associated with AD.^3^, ^4^, ^5^, ^6^, ^7^ PM_2.5_ is a mixture of tiny solid and liquid particles suspended in the air. PM_2.5_ is considered especially harmful to human health because they can enter deeper into our bodies.^8^ Outdoor sources of PM_2.5_ are fossil fuel combustion by automobiles, power plants, and industrial processes, while indoor sources are tobacco smoking, cooking, candle burning, and wood stove burning.^8^

Developing fetus during the prenatal period are susceptible to the effects of environmental exposure because their immune and organ systems are developing.^9^ Prenatal PM_2.5_ exposure and maternal stress induce oxidative stress,^10^, ^11^ one of the important mechanisms of AD pathogenesis.^12^, ^13^ Epigenetic modifications mediate the effect of various environmental exposures on allergic diseases.^14^ Prenatal PM_2.5_ and maternal stress have also been shown to modulate DNA methylation in the placenta or cord blood of newborns.^15^, ^16^, ^17^ Thus, prenatal PM_2.5_ exposure and maternal stress may have synergistic effects on disease in infants. Several studies have shown the interactive effect of PM_2.5_ and prenatal stress on asthma in children.^18^, ^19^ However, the combined effect of these exposures on the risk of AD in offspring has not been examined. Furthermore, environmental factors may affect the disease development depending on the time of exposure during fetal development. The critical exposure periods for the interactive effect of prenatal PM_2.5_ exposure and maternal stress also have not been examined in relation to infant AD.

PM_2.5_ and psychosocial stress may also have gender‐specific effects on the outcomes of the offspring. Boys were shown to be more vulnerable to prenatal PM_2.5_ exposure and maternal stress in terms of asthma development.^18^, ^20^ Girls with higher perinatal stress had increased risks of AD compared to that in boys.^21^ The gender‐specific effects of prenatal PM_2.5_ and maternal stress exposure during pregnancy on the offspring’s AD and their critical exposure periods remain unknown.

Other environmental factors also co‐vary with the effect of PM_2.5_ and/or stress on disease in children. Indoor PM_2.5_ is a significant exposure, especially for pregnant women who spend most of their time indoors. Antioxidative diets may have a mitigating effect of PM_2.5_ and stress. However, previous studies did not adjust for indoor PM_2.5_ and antioxidative diets.^6^, ^18^, ^19^ Several studies have assessed maternal distress by assessing major life events.^18^, ^19^ However, because major life events are infrequent, they are limited in assessing maternal stress during pregnancy.

We, thus, adjusted indoor PM_2.5_ and antioxidative diet during pregnancy to overcome these limitations of prior studies. The aim of this study is to investigate the combined effects of prenatal PM_2.5_ exposure and maternal anxiety during pregnancy on the risk of AD in infancy, including the different effects according to infant gender. Moreover, we also aimed to identify the critical period of PM_2.5_ exposure for AD in relation to maternal anxiety and infant gender.

## 2 METHODS

### 2.1 Study design and study population

The COhort for Childhood Origin of Asthma and allergic diseases (COCOA) study is a prospective birth cohort that aims to identify various environmental factors for childhood allergic diseases. The study has been described in detail elsewhere.^22^ Of the 1869 infants who were followed up for the first year, 799 had no data on indoor PM_2.5_ during pregnancy and 268 lacked data on prenatal maternal anxiety or diet. Therefore, this study included a total of 802 infants.

The present study protocol was approved by the institutional review boards of Asan Medical Center (IRB No. 2008‐0616), Samsung Medical Center (IRB No. 2009‐02‐021), Yonsei University (IRB No. 4‐2008‐0588), CHA Medical Center (IRB No. 2010‐010), and Seoul National University Hospital (IRB No. H‐1401‐086‐550). Written informed consent was obtained from the parents of each child.

### 2.2 Exposure and outcome measures

AD was determined from parental reports of a physician diagnosis of AD at the 6‐ and 12‐month follow‐up visits. We estimated exposure to outdoor PM_2.5_ using land‐use regression (LUR) models. We used ambient concentrations of PM_2.5_ measured by the Korean Ministry of Environment (http://www.airkorea.or.kr/web) at 37 fixed monitoring stations in the study area (Seoul). We used centrally and locally available geographic variables as a potential predictors. Predictor variables, such as traffic indicators, surrounding land usage, topography, and spatial trends, were computed at each location using ArcGIS version 9.3 (ESRI). Multiple linear regression models were built using a supervised forward stepwise procedure. Predictor variables used in the final LUR model for air pollution included the lengths of all roads, traffic intensity on the nearest road, total heavy‐duty traffic loads for all roads, and variables representing spatial trends. The models explained 66%–69% of the variability in measured PM_2.5_ levels and the predicted values fitted well with the measured values, as reported in our previous study.^23^

Indoor PM_2.5_ levels were measured by specialists during home visits occurring between weeks 26 and 36 of pregnancy. Indoor PM_2.5_ levels were measured three times in the parents’ bedroom by using a particle discriminator (Model GT‐331; SIBATA Co.) with a laser light‐scattering optical particle counter for 5 min. The mean value of three measurements was used for evaluation.^24^

Maternal anxiety was assessed by self‐reported questionnaires at 36 weeks of pregnancy based on the State‐Trait Anxiety Inventory‐Trait subscale (STAI‐T). The STAI‐T is a 20‐item questionnaire that is scored on a 4‐point Likert‐type scale that reflects a general tendency to be anxious. The score ranges from 20 to 80, with a higher score indicating more severe anxiety. In this study, subjects with scores above the 25th percentile (STAI = 46) were categorized as being anxious. The Korean version of STAI was previously shown to exhibit excellent psychometric properties. In terms of internal consistency, the reported Cronbach’s α coefficient is 0.91. In this study, the reliability coefficient of STAI was 0.92.^6^

### 2.3 Statistical analysis

The associations between PM_2.5_ exposure during each trimester of pregnancy and AD at 1 year of age were evaluated with a logistic regression model. The prenatal period was divided into three trimesters as follows: weeks 1–13 (first), weeks 14–27 (second), and weeks 28–40 (third). The PM_2.5_ levels were dichotomized as high or low according to the median value; these dichotomized values were used in the logistic regression analysis. The results are expressed as adjusted odds ratios (aORs) and 95% confidence intervals (CIs).

A Bayesian distributed lag interaction model was implemented to determine the critical windows during the prenatal period for the effects of prenatal PM_2.5_ in relation to AD at 1 year of age. The significant critical windows were identified as the weeks during pregnancy with statistically significant associations, as previously described.^25^ The models were adjusted for potential confounders, including a family history of allergic diseases, maternal education, pet ownership during pregnancy, intake of antioxidants during pregnancy (the sum of daily intakes of antioxidants, such as vitamin A, vitamin C, vitamin E, retinol, and carotene), secondhand smoking during pregnancy, indoor PM_2.5_ during pregnancy, birth season, infant gender, and breastfeeding until 6 months. The analyses were performed using SAS version 9.4 and R statistical software (v3.6.1), with *p* < 0.05 is considered as statistically significant.

## 3 RESULTS

### 3.1 Characteristics of the study population

In this study, 107 (13.3%) infants were diagnosed with AD at 1 year. Significantly more boys than girls were diagnosed with AD (*p* = 0.03). Family history of allergic diseases was higher in infants with AD than in those without AD (*p* = 0.03). The other characteristics of the study participants did not differ significantly between those with and without AD at 1 year (Table S1).

### 3.2 Associations between prenatal PM_2.5_ exposure, maternal anxiety during pregnancy, and infant gender with AD at 1 year of age

The association between PM_2.5_ during the first trimester of pregnancy and AD at 1 year of age was significant (aOR: 1.86; 95% CI: 1.08–3.19). PM_2.5_ exposure during the entire pregnancy, second and third trimesters of pregnancy were not associated with AD at 1 year of age (Table 1). AD at 1 year of age increased in infants exposed to higher maternal anxiety during pregnancy and boys (aOR: 1.58; 95% CI: 1.07–2.47 and aOR: 1.54; 95% CI: 1.01–2.36; Table 1).

**TABLE 1:** Associations between prenatal PM_2.5_ exposure, maternal anxiety during pregnancy, and gender with AD at 1 year of age

	aOR^a^ (95% CI)
Boy	1.54 (1.01–2.36)
Higher STAI	1.58 (1.01–2.47)
Higher PM_2.5_ during the entire pregnancy	0.99 (0.64–1.53)
Higher PM_2.5_ during the first trimester	1.86 (1.08–3.19)
Higher PM_2.5_ during the second trimester	0.84 (0.51–1.41)
Higher PM_2.5_ during the third trimester	0.62 (0.37–1.04)

Abbreviations: AD, atopic dermatitis; aOR, adjusted odds ratio; CI, confidence interval; PM, particulate matter; STAI, State‐Trait Anxiety Inventory.

^a^ Adjusted for family history of allergy diseases, maternal education, pet ownership during pregnancy, antioxidant intake during pregnancy, secondhand smoking during pregnancy, birth season, breastfeeding, and indoor PM_2.5_ exposure at any time during weeks 26–36.

### 3.3 Combined effect of prenatal PM_2.5_ exposure and maternal anxiety during pregnancy on AD at 1 year of age

Infants with both higher PM_2.5_ during the first trimester of pregnancy and higher maternal anxiety during pregnancy showed an increased incidence of AD at 1 year of age (aOR: 3.13; 95% CI: 1.56–6.28, interaction *p*‐value = 0.35; Table 2).

**TABLE 2:** Associations between prenatal PM_2.5_ exposure and maternal anxiety during pregnancy with AD at 1 year of age

		aOR^a^ (95% CI)	Interaction *p*‐value
PM_2.5_ during entire pregnancy			
STAI (low)	PM_2.5_ (low)	Reference	0.27
STAI (low)	PM_2.5_ (high)	1.20 (0.71–2.02)	
STAI (high)	PM_2.5_ (low)	2.06 (1.12–3.81)	
STAI (high)	PM_2.5_ (high)	1.43 (0.73–2.80)	
PM_2.5_ during the first trimester			
STAI (low)	PM_2.5_ (low)	Reference	0.35
STAI (low)	PM_2.5_ (high)	1.65 (0.89–3.07)	
STAI (high)	PM_2.5_ (low)	1.29 (0.66–2.54)	
STAI (high)	PM_2.5_ (high)	3.13 (1.56–6.28)	
PM_2.5_ during the second trimester			
STAI (low)	PM_2.5_ (low)	Reference	0.41
STAI (low)	PM_2.5_ (high)	0.76 (0.42–1.37)	
STAI (high)	PM_2.5_ (low)	1.30 (0.69–2.45)	
STAI (high)	PM_2.5_ (high)	1.45 (0.73–2.87)	
PM_2.5_ during the third trimester			
STAI (low)	PM_2.5_ (low)	Reference	0.21
STAI (low)	PM_2.5_ (high)	0.76 (0.42–1.38)	
STAI (high)	PM_2.5_ (low)	2.05 (1.13–3.71)	
STAI (high)	PM_2.5_ (high)	0.88 (0.42–1.83)	

Abbreviations: AD, atopic dermatitis; aOR, adjusted odds ratio; CI, confidence interval; PM, particulate matter; STAI, State‐Trait Anxiety Inventory.

^a^ Adjusted for family history of allergy diseases, maternal education, pet ownership during pregnancy, antioxidant intake during pregnancy, secondhand smoking during pregnancy, birth season, infant gender, breastfeeding, and indoor PM_2.5_ exposure at any time during weeks 26–36.

### 3.4 Modifying effect of infant gender on the effects of PM_2.5_ and maternal anxiety on AD at 1 year of age

Boys with higher PM_2.5_ during the first trimester of pregnancy showed an increased risk of AD at 1 year of age (aOR: 2.33; 95% CI: 1.10–4.96; Table 3). Higher maternal anxiety during pregnancy was associated with an increased AD at 1 year of age in girls (aOR: 3.21; 95% CI: 1.61–6.39; Table 3). The combined effect of higher PM_2.5_ exposure during the first trimester and higher maternal anxiety was significant only in boys (aOR: 5.30; 95% CI: 1.14–24.65; Table 4).

**TABLE 3:** Associations between prenatal PM_2.5_ exposure and maternal anxiety during pregnancy with AD at 1 year of age according to infant gender

	aOR^a^ (95% CI)	
	Boys	Girls
Higher STAI	0.92 (0.49–1.71)	3.21 (1.61–6.39)
Higher PM_2.5_ during the entire pregnancy	0.98 (0.55–1.73)	0.96 (0.48–1.92)
Higher PM_2.5_ during the first trimester	2.33 (1.10–4.96)	1.48 (0.66–3.31)
Higher PM_2.5_ during the second trimester	0.83 (0.44–1.58)	0.80 (0.34–1.91)
Higher PM_2.5_ during the third trimester	0.62 (0.31–1.23)	0.63 (0.28–1.38)

Abbreviations: AD, atopic dermatitis; aOR, adjusted odds ratio; CI, confidence interval; PM, particulate matter; STAI, State‐Trait Anxiety Inventory.

^a^ Adjusted for family history of allergy diseases, maternal education, pet ownership during pregnancy, antioxidant intake during pregnancy, secondhand smoking during pregnancy, birth season, breastfeeding, and indoor PM_2.5_ exposure at any during weeks 26–36.

**TABLE 4:** Associations between prenatal PM_2.5_ exposure and AD according to infant gender and maternal anxiety during pregnancy

Higher PM_2.5_	aOR^a^ (95% CI)			
	Boys with higher STAI	Boys with lower STAI	Girls with higher STAI	Girls with lower STAI
Entire pregnancy	0.66 (0.21–2.04)	1.12 (0.57–2.19)	0.67 (0.21–2.12)	1.65 (0.63–4.29)
First trimester	5.30 (1.14–24.65)	1.82 (0.73–4.55)	2.07 (0.57–7.44)	1.35 (0.43–4.24)
Second trimester	1.09 (0.32–3.71)	0.81 (0.38–1.74)	1.71 (0.38–7.62)	0.57 (0.16–1.98)
Third trimester	0.08 (0.01–0.53)	0.95 (0.44–2.06)	0.79 (0.23–2.71)	0.61 (0.20–1.86)

Abbreviations: AD, atopic dermatitis; aOR, adjusted odds ratio; CI, confidence interval; PM, particulate matter; STAI, State‐Trait Anxiety Inventory.

^a^ Adjusted for family history of allergy diseases, maternal education, pet ownership during pregnancy, antioxidant intake during pregnancy, secondhand smoking during pregnancy, birth season, breastfeeding, and indoor PM_2.5_ exposure at any time during weeks 26–36.

### 3.5 Critical periods of prenatal PM_2.5_ exposure on AD at 1 year of age according to infant gender and maternal anxiety during pregnancy

**FIGURE 1:**
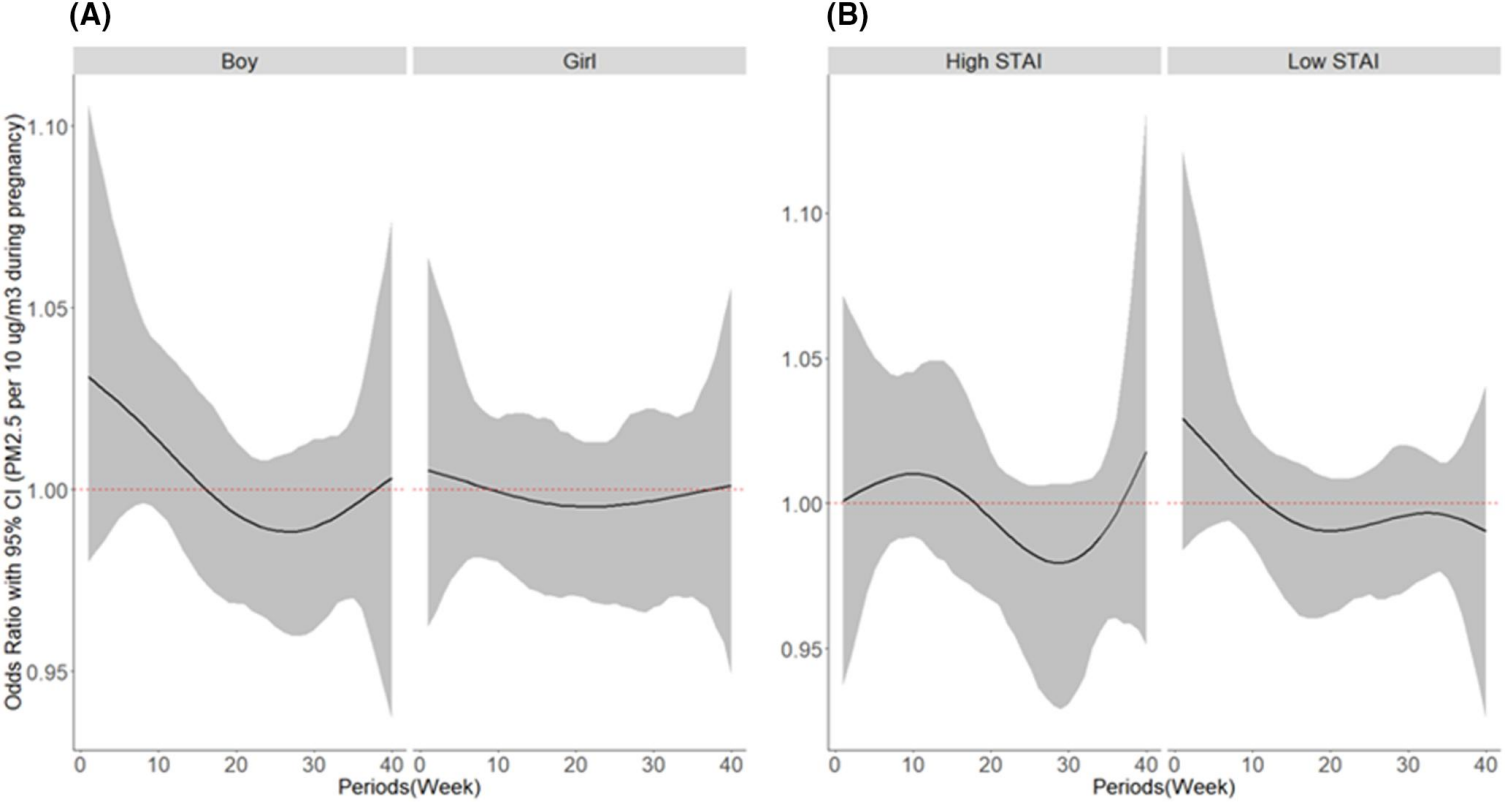
Associations between prenatal PM_2.5_ exposure and atopic dermatitis (AD) at 1 year of age according to (A) infant gender and (B) maternal anxiety during pregnancy. Bayesian distributed lag interaction model was used to estimate the critical period for the association between PM_2.5_ exposure over pregnancy and AD according to (A) infant gender and (B) prenatal maternal anxiety (high vs. low). The models were adjusted for family history of allergic diseases, maternal education, pet ownership during pregnancy, intake of antioxidants during pregnancy (the sum of daily intakes of antioxidants, such as vitamin A, vitamin C, vitamin E, retinol, and carotene), secondhand smoking during pregnancy, indoor PM_2.5_ during pregnancy, birth season, infant gender, and breastfeeding until 6 months. The *y*‐axis represents the odds ratio (OR) of AD in relation to PM_2.5_ exposure. The *x*‐axis represents the gestational age in weeks. The black solid line represents the predicted OR, with the gray area indicating the 95% confidence interval (CI). A sensitive window was defined as that with an estimated pointwise 95% CI not including zero. PM_2.5_, particulate matter with an aerodynamic diameter of <2.5 μm; STAI, State‐Trait Anxiety Inventory

**FIGURE 2:**
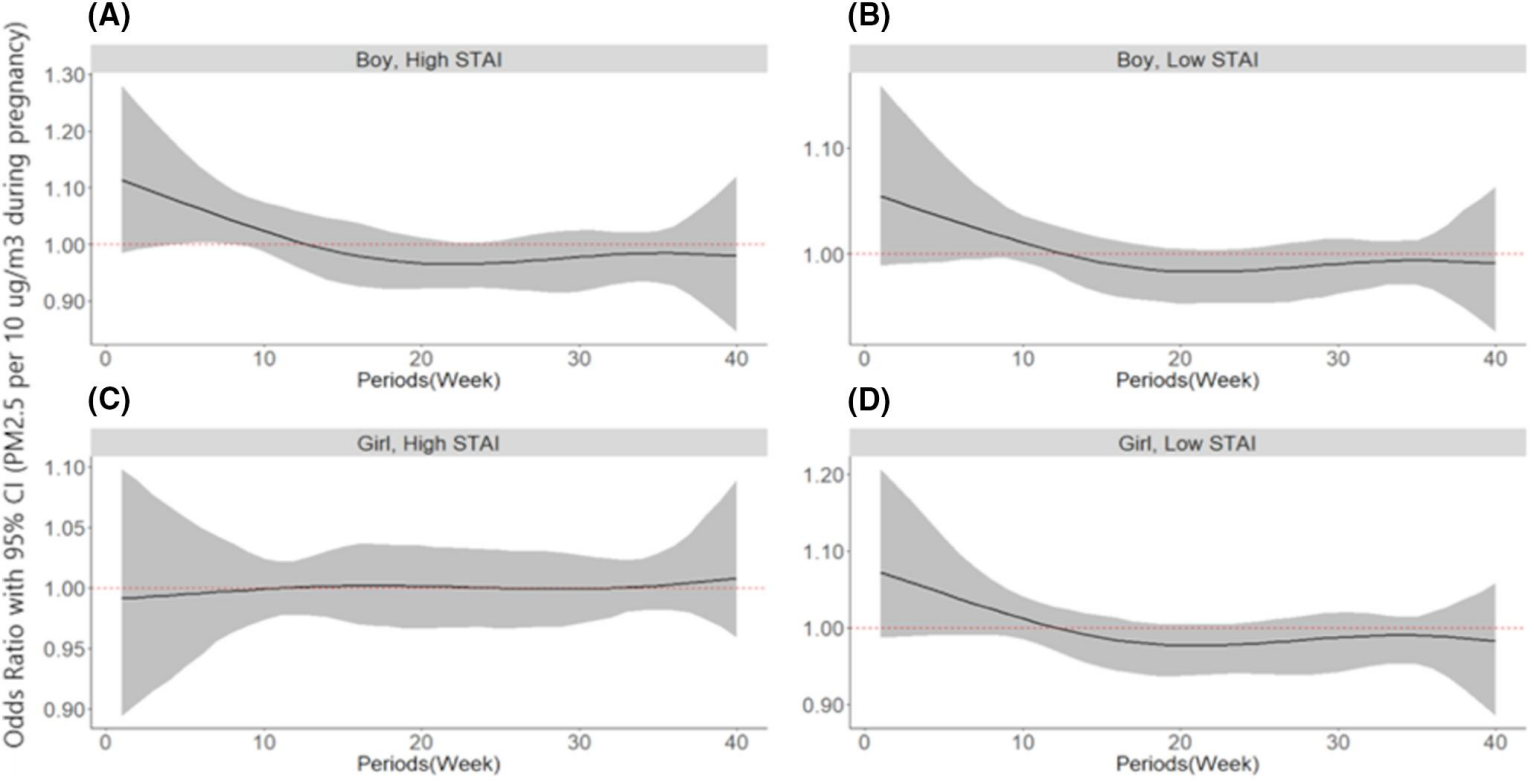
Associations between prenatal PM_2.5_ exposure and atopic dermatitis (AD) at 1 year of age stratified by infant gender and maternal anxiety during pregnancy. Bayesian distributed lag interaction model was used to estimate the critical period for the association between PM_2.5_ exposure over pregnancy and AD stratified by infant gender and prenatal maternal anxiety (high vs. low); (A) boys with higher prenatal maternal anxiety, (B) boys with lower prenatal maternal anxiety, (C) girls with higher prenatal maternal anxiety, and (D) girls with lower prenatal maternal anxiety. The models were adjusted for family history of allergic diseases, maternal education, pet ownership during pregnancy, intake of antioxidants during pregnancy (the sum of daily intakes of antioxidants, such as vitamin A, vitamin C, vitamin E, retinol, and carotene), secondhand smoking during pregnancy, indoor PM_2.5_ during pregnancy, birth season, and breastfeeding until 6 months. The *y*‐axis represents the odds ratio (OR) of AD in relation to PM_2.5_ exposure. The *x*‐axis represents the gestational age in weeks. The black solid line represents predicted OR, with the gray area indicating the 95% confidence interval (CI). A sensitive window was defined as that with an estimated pointwise 95% CI not including zero. PM_2.5_, particulate matter with an aerodynamic diameter of <2.5 μm; STAI, State‐Trait Anxiety Inventory

There were no critical PM_2.5_ exposure periods for AD at 1 year of age in either boys or girls (Figure 1A). No critical PM_2.5_ exposure periods for AD at 1 year of age were identified in infants with both higher and lower maternal anxiety levels during pregnancy (Figure 1B). However, gestation weeks 5–8 were critical periods of PM_2.5_ exposure affecting AD at 1 year of age in boys with higher maternal anxiety during pregnancy (Figure 2).

## 4 DISCUSSION

We evaluated the effect of prenatal PM_2.5_ exposures and maternal anxiety on AD at 1 year of age by adjusting indoor PM_2.5_ and antioxidative diet during pregnancy. We identified the independent effects of PM_2.5_ exposure during the first trimester of pregnancy and maternal anxiety during pregnancy on the risk of AD. Moreover, infants who were concurrently exposed to higher PM_2.5_ during the first trimester of pregnancy and higher maternal anxiety during pregnancy showed increased risks of AD. Boys were more vulnerable to co‐exposure of PM_2.5_ and maternal anxiety compared to girls. PM_2.5_ exposure during gestation weeks 5–8 was associated with AD in boys with higher maternal anxiety during pregnancy. These results suggested that the development of AD in early life may be affected by exposure to PM_2.5_ during the first trimester of pregnancy, especially in boys with higher maternal distress. Avoidance of PM_2.5_ exposure and maternal anxiety during the first trimester of pregnancy is critical to prevent AD development in early life.

Exposure to PM_2.5_ during the prenatal period is associated with childhood AD. PM_2.5_ exposure during gestation weeks 7–17 was associated with increased childhood eczema.^3^ This result is consistent with ours regarding the association between increased AD at 1 year of age and PM_2.5_ exposure during the first trimester of pregnancy.

In addition to prenatal exposure to PM, prenatal maternal stress is also associated with AD in the offspring. A recent systematic review showed that prenatal maternal stress, especially during the third trimester, was associated with an increased risk of AD in the offspring.^26^ Our finding that prenatal maternal anxiety, as assessed with a questionnaire at 36 weeks gestation, was associated with increase AD in the offspring was consistent with that of the systematic review.

Maternal exposure to PM_2.5_ and psychosocial stress during pregnancy are associated with oxidative stress.^10^, ^11^, ^27^ Oxidative stress may influence AD pathogenesis. Oxidative stress during pregnancy may affect fetal development and growth.^10^, ^27^ Oxidative stress could also modulate T‐cell polarization toward a subset of T‐helper 2 (Th2) cells, alter cytokine release and induce damage in keratinocytes.^13^

Oxidative stress can be affected differently according to gender. Estrogen is associated with cellular responses to oxidative stress, suggesting its protective role.^28^ The levels of oxidative stress markers are higher and the markers related to antioxidant capacity are lower in boys compared to girls during the neonatal period.^29^ These gender differences in oxidative stress may explain our findings that boys were more susceptible to prenatal PM_2.5_ and maternal anxiety related to early‐onset AD.

Epigenetic mechanisms could explain the effect of prenatal environmental factors on disease development in the offspring.^14^ Prenatal exposure to PM_2.5_ influences placental adaptation and fetal immune development by DNA methylation.^15^ Maternal distress during pregnancy can also alter DNA methylation, causing increased allergy risk in newborns.^17^, ^30^ The vulnerable gender for epigenetic changes by prenatal PM_2.5_ and maternal stress has not yet been identified. Furthermore, study is needed to evaluate gender‐specific epigenetic changes and whether they are related to later differences in disease development between boys and girls.

As both prenatal PM_2.5_ and maternal stress may have common mechanistic pathways, co‐exposures of PM_2.5_ and maternal stress may affect AD in infants. While the interaction between prenatal PM_2.5_ and maternal stress has been shown in childhood asthma,^18^, ^19^ no study has examined the interaction between these factors in relation to childhood AD. In our study, co‐exposure to PM_2.5_ and higher maternal anxiety during pregnancy increased AD at 1 year of age in boys but not girls. Our results suggest that prenatal PM_2.5_ and prenatal maternal anxiety have combined effect on the risk of AD in the offspring and that boys are more vulnerable to these effects.

Our finding that the first trimester of pregnancy was a critical period for PM_2.5_ exposure associated with AD at 1 year of age was consistent with those of previous COCOA studies.^4^, ^5^ In particular, 5–8 weeks of gestation was the critical period for PM_2.5_ exposure in boys with higher prenatal maternal anxiety. The development of antioxidant enzyme systems begins during the mid‐to‐late gestational periods, and these systems continue to mature through the early postnatal years.^31^ Thus, before these enzyme systems are established, infants in early pregnancy are more susceptible to oxidative stress from PM_2.5_ and stress. PM_2.5_ exposure could significantly impact placental DNA methylation patterns from the first trimester of pregnancy.^15^ Alterations of DNA methylation in the placenta influence placental development. As placental function is important in fetal programing, placental DNA methylation is associated with abnormal fetal development.^32^ In particular, the fetal skin structure develops rapidly during the first trimester of pregnancy.^33^ Therefore, the first trimester of pregnancy, especially gestational weeks 5–8, is a critical period for the effects of PM_2.5_ exposure on AD.

Our study has several strengths. First, it is a prospective general population‐based birth cohort study; thus, we can generalize our results to the general population. Second, we adjusted the data for not only common confounding factors but also indoor PM_2.5_ and antioxidant intake during pregnancy. Therefore, we could more accurately identify the effect of prenatal PM_2.5_ exposure and the critical periods of these effects on AD in the offspring. Lastly, we used valid self‐reported questionnaires that provide suitable measures of usual distress during pregnancy. Therefore, it may be more appropriate to measure maternal depression or anxiety in usual life.

Our study has several limitations. First, we used exposure modeling to estimate the concentrations of PM_2.5_, a method widely used in epidemiologic studies to estimate exposure levels.^5^, ^33^ Some misclassifications of PM_2.5_ exposure were possible with this system; however, it is not feasible to use individual and continuous direct pollutant monitoring in a large general population. Second, data on prenatal maternal anxiety were collected with questionnaires (STAI) at 36 weeks of pregnancy. Therefore, it may not reflect maternal anxiety during the entire pregnancy. A systematic review showed consistent results with our findings that the third trimester of pregnancy was a more vulnerable period for stress exposure compared to the first and second trimesters.^26^ However, there have been no studies using STAI in several times during pregnancy to investigate the effect of prenatal maternal anxiety on offspring’s AD. Furthermore, studies using STAI in several times during pregnancy may help to identify the critical period of prenatal anxiety exposure for AD in offspring. Third, we did not adjust for other ambient air pollution. However, we adjusted for secondhand smoke and indoor PM_2.5_, which we believe to be more relevant for pregnant women who spend most of their time indoors. Fourth, because indoor PM_2.5_ measurements started later in the COCOA study, this factor was not measured in all study participants. Maternal diet and anxiety during pregnancy were also not evaluated in all study participants. Therefore, selection bias was possible in this study.

The findings of the present study suggested that PM_2.5_ exposure during the first trimester of pregnancy and maternal anxiety during pregnancy increased the probability of AD at 1 year of age. Boys born to mothers exposed to both increased PM_2.5_ during weeks 5–8 of gestation and anxiety during pregnancy were at increased risk for AD at 1 year of age. Avoidance of exposure to PM_2.5_ and maternal anxiety during the prenatal period, especially in the first trimester, may prevent the development of infantile AD.

## CONFLICT OF INTEREST

The authors acknowledge that there is no conflict of interest to declare.

## Supporting information

Supplementary Material

## References

[1] Weidinger S , Novak N . Atopic dermatitis. Lancet. 2016;387(10023):1109‐1122. 26377142 10.1016/S0140-6736(15)00149-X

[2] Lee JY , Seo JH , Kwon JW , et al. Exposure to gene‐environment interactions before 1 year of age may favor the development of atopic dermatitis. Int Arch Allergy Immunol. 2012;157(4):363‐371. 22123373 10.1159/000328778

[3] Yao TC , Huang HY , Pan WC , et al. Association of prenatal exposure to fine particulate matter pollution with childhood eczema. Allergy. 2021;76(7):2241‐2245. 33432626 10.1111/all.14738 PMC8249326

[4] Yang SI , Lee SH , Lee SY , et al. Prenatal PM_2.5_ exposure and vitamin D‐associated early persistent atopic dermatitis via placental methylation. Ann Allergy Asthma Immunol. 2020;125(6):665‐673. 32971247 10.1016/j.anai.2020.09.008

[5] Lee E , Lee SY , Kim HC , et al. Prenatal particulate matter exposure with skin barrier dysfunction affects offspring’s atopic dermatitis: COCOA study. J Allergy Clin Immunol Pract. 2020;8(6):2062‐2065. 32006726 10.1016/j.jaip.2020.01.040

[6] Chang HY , Suh DI , Yang SI , et al. Prenatal maternal distress affects atopic dermatitis in offspring mediated by oxidative stress. J Allergy Clin Immunol. 2016;138(2):468‐475. 27016803 10.1016/j.jaci.2016.01.020

[7] El‐Heis S , Crozier SR , Healy E , et al. Maternal stress and psychological distress preconception: association with offspring atopic eczema at age 12 months. Clin Exp Allergy. 2017;47(6):760‐769. 28218994 10.1111/cea.12910 PMC5447366

[8] Kelly FJ , Fussell JC . Size, source and chemical composition as determinants of toxicity attributable to ambient particulate matter. Atmos Environ. 2012;60:504‐526.

[9] Lockett GA , Huoman J , Holloway JW . Does allergy begin in utero? Pediatr Allergy Immunol. 2015;26(5):394‐402. 26011578 10.1111/pai.12408

[10] Grevendonk L , Janssen BG , Vanpoucke C , et al. Mitochondrial oxidative DNA damage and exposure to particulate air pollution in mother‐newborn pairs. Environ Health. 2016;15:10. 26792633 10.1186/s12940-016-0095-2 PMC4719654

[11] Brunst KJ , Sanchez Guerra M , Gennings C , et al. Maternal lifetime stress and prenatal psychological functioning and decreased placental mitochondrial DNA copy number in the PRISM study. Am J Epidemiol. 2017;186(11):1227‐1236. 28595325 10.1093/aje/kwx183 PMC5859981

[12] Ahn K . The role of air pollutants in atopic dermatitis. J Allergy Clin Immunol. 2014;134(5):993‐999. 25439225 10.1016/j.jaci.2014.09.023

[13] Suh DI , Chang HY , Lee E , Yang SI , Hong SJ . Prenatal maternal distress and allergic diseases in offspring: review of evidence and possible pathways. Allergy Asthma Immunol Res. 2017;9(3):200‐211. 28293926 10.4168/aair.2017.9.3.200 PMC5352571

[14] Potaczek DP , Harb H , Michel S , Alhamwe BA , Renz H , Tost J . Epigenetics and allergy: from basic mechanisms to clinical applications. Epigenomics. 2017;9(4):539‐571. 28322581 10.2217/epi-2016-0162

[15] Maghbooli Z , Hossein‐Nezhad A , Adabi E , et al. Air pollution during pregnancy and placental adaptation in the levels of global DNA methylation. PLoS One. 2018;13(7):e0199772. 29979694 10.1371/journal.pone.0199772 PMC6034814

[16] Wright RJ . Perinatal stress and early life programming of lung structure and function. Biol Psychol. 2010;84(1):46‐56. 20080145 10.1016/j.biopsycho.2010.01.007 PMC2888999

[17] Devlin AM , Brain U , Austin J , Oberlander TF . Prenatal exposure to maternal depressed mood and the MTHFR C677T variant affect SLC6A4 methylation in infants at birth. PLoS One. 2010;5(8):e12201. 20808944 10.1371/journal.pone.0012201 PMC2922376

[18] Lee A , Leon Hsu HH , Mathilda Chiu YH , et al. Prenatal fine particulate exposure and early childhood asthma: effect of maternal stress and fetal sex. J Allergy Clin Immunol. 2018;141(5):1880‐1886. 28801196 10.1016/j.jaci.2017.07.017 PMC5803480

[19] Rosa MJ , Just AC , Kloog I , et al. Prenatal particulate matter exposure and wheeze in Mexican children: effect modification by prenatal psychosocial stress. Ann Allergy Asthma Immunol. 2017;119(3):232‐237. 28757229 10.1016/j.anai.2017.06.016 PMC5593766

[20] Lee A , Mathilda Chiu YH , Rosa MJ , et al. Prenatal and postnatal stress and asthma in children: temporal‐ and sex‐specific associations. J Allergy Clin Immunol. 2016;138(3):740‐747. 26953156 10.1016/j.jaci.2016.01.014 PMC5011027

[21] Yoon J , Kim EM , Lee MY , et al. Perinatal maternal negative life events as risk factors of atopic dermatitis in female offspring. Ann Allergy Asthma Immunol. 2018;121(5):641‐642. 10.1016/j.anai.2018.07.020 30036583

[22] Yang HJ , Lee SY , Suh DI , et al. The Cohort for Childhood Origin of Asthma and allergic diseases (COCOA) study: design, rationale and methods. BMC Pulm Med. 2014;14:109. 24990471 10.1186/1471-2466-14-109 PMC4099383

[23] Lamichhane DK , Leem JH , Kim HC . Associations between ambient particulate matter and nitrogen dioxide and chronic obstructive pulmonary diseases in adults and effect modification by demographic and lifestyle factors. Int J Environ Res Public Health. 2018;15(2):363. 10.3390/ijerph15020363 PMC5858432 29463050

[24] Yang SI , Kim BJ , Lee SY , et al. Prenatal particulate matter/tobacco smoke increases infants’ respiratory infections: COCOA study. Allergy Asthma Immunol Res. 2015;7(6):573‐582. 26333704 10.4168/aair.2015.7.6.573 PMC4605930

[25] Wilson A , Chiu YM , Hsu HL , Wright RO , Wright RJ , Coull BA . Bayesian distributed lag interaction models to identify perinatal windows of vulnerability in children’s health. Biostatistics. 2017;18(3):537‐552. 28334179 10.1093/biostatistics/kxx002 PMC5862289

[26] Flanigan C , Sheikh A , DunnGalvin A , Brew BK , Almqvist C , Nwaru BI . Prenatal maternal psychosocial stress and offspring’s asthma and allergic disease: a systematic review and meta‐analysis. Clin Exp Allergy. 2018;48(4):403‐414. 29331049 10.1111/cea.13091

[27] Saenen ND , Vrijens K , Janssen BG , et al. Placental nitrosative stress and exposure to ambient air pollution during gestation: a population study. Am J Epidemiol. 2016;184(6):442‐449. 27601048 10.1093/aje/kww007

[28] Lavoie JC , Tremblay A . Sex‐specificity of oxidative stress in newborns leading to a personalized antioxidant nutritive strategy. Antioxidants. 2018;7(4):49. 10.3390/antiox7040049 PMC5946115 29584624

[29] Mueller BR , Bale TL . Sex‐specific programming of offspring emotionality after stress early in pregnancy. J Neurosci. 2008;28(36):9055‐9065. 18768700 10.1523/JNEUROSCI.1424-08.2008 PMC2731562

[30] Oberlander TF , Weinberg J , Papsdorf M , Grunau R , Misri S , Devlin AM . Prenatal exposure to maternal depression, neonatal methylation of human glucocorticoid receptor gene (NR3C1) and infant cortisol stress responses. Epigenetics. 2008;3(2):97‐106. 18536531 10.4161/epi.3.2.6034

[31] Wright RJ , Brunst KJ . Programming of respiratory health in childhood: influence of outdoor air pollution. Curr Opin Pediatr. 2013;25(2):232‐239. 23422354 10.1097/MOP.0b013e32835e78cc

[32] Saenen ND , Martens DS , Neven KY , et al. Air pollution‐induced placental alterations: an interplay of oxidative stress, epigenetics, and the aging phenotype? Clin Epigenet. 2019;11(1):124. 10.1186/s13148-019-0688-z PMC6749657 31530287

[33] Lee JY , Lamichhane DK , Lee M , et al. Preventive effect of residential green space on infantile atopic dermatitis associated with prenatal air pollution exposure. Int J Environ Res Public Health. 2018;15(1):102. 10.3390/ijerph15010102 PMC5800201 29315266

