# Supplementary material for: Prenatal PM2.5 affects atopic dermatitis depending on maternal anxiety and gender: COCOA study
Source: Clin Transl Allergy. 2021 Oct 15;11(8):e12070. doi: 10.1002/clt2.12070 (PMC8519998; doi:10.1002/clt2.12070)
Supplement: Supplementary file 1 — Supplementary Material [file CLT2-11-e12070-s001.docx]

**TableE1. Characteristics of the study population**

|  |  | Total  (N=802) | AD at 1 year of age (+)  (n=107) | AD at 1 year of age (-)  (n=695) | P-value |
| --- | --- | --- | --- | --- | --- |
| Sex (boys) |  | 420 (52.4) | 66 (61.7) | 354 (50.9) | 0.03 |
| Birth weight (g) |  | 3207.7 ± 416.2 | 3164.7 ± 373.0 | 3214.2 ± 422.2 | 0.29 |
| Gestational age (weeks) |  | 39.3 ± 1.1 | 39.3 ± 1.0 | 39.3 ± 1.1 | 0.89 |
| Family history of allergic disease |  | 441 (55.0) | 69 (64.5) | 372 (53.5) | 0.03 |
| Birth season | Spring | 208 (25.9) | 32 (29.9) | 176 (25.3) | 0.69 |
|  | Summer | 171 (21.3) | 20 (18.7) | 151 (21.7) |  |
|  | Fall | 179 (22.3) | 25 (23.4) | 154 (22.2) |  |
|  | Winter | 244 (30.4) | 30 (28.0) | 214 (30.8) |  |
| Maternal age at delivery (years) |  | 33.8 ± 3.6 | 34.3 ± 3.4 | 33.7 ± 3.6 | 0.11 |
| Maternal education | High school | 39 (4.9) | 4 (3.7) | 35 (5.0) | 0.63 |
|  | University | 585 (72.9) | 76 (71.0) | 509 (73.2) |  |
|  | Graduate school | 178 (22.2) | 27 (25.3) | 151 (21.8) |  |
| Family income  (Korean million won) | < 3 | 154 (19.3) | 20 (18.7) | 134 (19.4) | 0.14 |
|  | 3 – 4 | 172 (21.5) | 21 (19.6) | 151 (21.8) |  |
|  | 4 – 5 | 152 (19.0) | 29 (27.1) | 123 (17.8) |  |
|  | ≥ 5 | 321 (40.2) | 37 (34.6) | 284 (41.0) |  |
| Breastfeeding until 6 months |  | 144 (18.0) | 17 (15.9) | 127 (18.3) | 0.54 |
| Secondhand smoking  during pregnancy |  | 456 (56.9) | 56 (52.3) | 400 (57.5) | 0.31 |
| Pet ownership during pregnancy |  | 49 (6.1) | 3 (2.8) | 46 (6.6) | 0.12 |
| Antioxidant intake during pregnancy |  | 37868.6 ± 246048.5 | 35908.4 ± 121207.2 | 38170.4 ± 260056.2 | 0.33 |
| Indoor PM_2.5_ at 36 weeks of gestation (µg/m^3^) |  | 6.4 ± 5.3 | 6.4 ± 5.2 | 6.4 ± 5.3 | 0.98 |

Numbers are number (%) or mean ± standard deviation.

PM_2.5_, particulate matter with an aerodynamic diameter of <2.5 μm; AD, atopic dermatitis
